# Circulating MicroRNAs as Quantitative Biomarkers for Diagnosis and Prognosis of Uveal Melanoma

**DOI:** 10.3389/fonc.2022.854253

**Published:** 2022-03-31

**Authors:** Wen-Da Zhou, Lei Shao, Li Dong, Rui-Heng Zhang, Yi-Fan Li, He-Yan Li, Hao-Tian Wu, Xu-Han Shi, Wen-Bin Wei

**Affiliations:** Beijing Tongren Eye Center, Beijing Key Laboratory of Intraocular Tumor Diagnosis and Treatment, Beijing Ophthalmology & Visual Sciences Key Lab, Medical Artificial Intelligence Research and Verification Key Laboratory of the Ministry of Industry and Information Technology, Beijing Tongren Hospital, Capital Medical University, Beijing, China

**Keywords:** prognostic, diagnostic, biomarker, microRNAs, uveal melanoma

## Abstract

For uveal melanoma (UM) patients, it is significant to establish diagnosis and prognosis evaluation systems through imaging techniques. However, imaging examinations are short of quantitative biomarkers and it is difficult to finish early diagnosis of UM. In order to discover new molecular biomarkers for the diagnosis and prognostic evaluation of UM, six circulating miRNAs (mir-132-3p, mir-21-5p, mir-34a-5p, mir-126-3p, mir-199a-3p, mir-214-3p) were chosen as candidates for independent validation. Validation of these miRNAs was performed in a cohort of 20 patients, including 10 spindle-shaped melanoma and 10 epithelioid cell melanoma, and 10 healthy donors. Then 5 patients with metastatic UM were included to validate the performance of miRNAs in advanced UM. Serum levels of miRNAs were determined using quantitative real-time PCR. We confirmed significantly higher levels of three miRNAs in serum of UM patients in comparison to healthy controls, and miR-199a-3p had the best performance (*p* < 0.0001; AUC = 0.985). MiR-214-3p and miR-21-5p were significantly upregulated in serum of epithelioid cell melanoma patients compared to spindle-shaped melanoma patients and miR-132-3p and, conversely, were significantly downregulated in serum of epithelioid cell melanoma patients. MiR-21-5p shows their best performance (*p* < 0.0001; AUC = 0.980). Both miR-199a-3p and miR-21-5p showed great performance in advanced UM. Significantly higher levels of miR-21-5p (*p* < 0.001) were found in serum of metastatic UM patients compared to patients with localized spindle-shaped melanoma, and significantly higher levels of miR-199a-3p (*p* < 0.001) were detected in serum of metastatic UM patients compared to healthy controls. Our preliminary data indicate promising diagnostic utility of circulating miR-199a-3p and promising prognostic utility of circulating miR-21-5p in both early and advanced UM patients.

## Introduction

Uveal melanoma (UM) is the most common intraocular malignancy in adults and arises from melanocytes in the iris, ciliary body, or choroid. The clinical diagnosis of uveal melanoma is based on the slit lamp and indirect ophthalmoscope. Experienced retina specialists have no difficulty in diagnosing typical uveal melanoma based on the morphology of the tumor (1). However, in some cases, the diagnosis of UM will be challenging, especially when it is complicated with retinal detachment, refractive interstitial opacity, and small melanoma, which needs imaging techniques including ultrasonography and magnetic resonance imaging (MRI) to confirm the diagnosis. Metastatic melanoma was the leading single cause of death, and 40%–50% of patients with UM die from metastatic disease. Patients with metastatic disease frequently receive various treatments including surgical resection, chemotherapy, immunotherapy, and targeted therapy, but these treatments produce relatively modest health benefits (2).In spite that several cytogenetic features have been proved to have great value to estimate the prognosis of patients with UM, these methods are invasive and labor-intensive (2, 3). It is of great clinical significance to obtain *a priori* information of a poor prognosis through a non-invasive way. In summary, the current non-invasive examinations, mainly imaging techniques, are difficult to diagnose and evaluate the prognosis of UM at an early stage and it also lacks quantitative biomarkers. Therefore, novel non-invasive biomarkers for diagnosis and prognosis evaluation in very early stage are urgently needed.

MicroRNAs (miRNA) are small, non-coding, single-stranded RNA molecules that posttranscriptionally regulate gene expression by inhibiting or inactivating target messenger RNAs (4, 5). MiRNA expression patterns are frequently dysregulated in human tumors (6–10), including uveal melanoma (11, 12). Previous studies have shown that circulating miRNAs, detected in plasma or serum, could be an ideal class of blood-based biomarkers for cancer detection (13). The serum microRNA expression profile of tumor patients is significantly different from that of healthy people, and even tumor subtypes can be distinguished based on the serum microRNA expression profile (14). Unlike imaging examination, serum microRNAs change before tumors cause morphological changes, which makes circulating microRNAs an ideal non-invasive biomarker that can screen for tumors at an early stage (15, 16). In uveal melanoma, most studies on miRNA biomarkers have paid attention to the analysis of tumor tissue, while recently some studies have reported the diagnostic potential of circulating miRNAs (17–19). However, the prognostic utility of circulating miRNAs in uveal melanoma still needs to be proved and the diagnostic utility needs to be validated on larger cohorts of patients. Such studies are of great significance because they may provide quantitative biomarkers for the early diagnosis and prognostic evaluation of UM.

According to literature review, we finally selected six candidate miRNAs (miR-132-3p, miR-21-5p, miR-34a-5p, miR-126-3p, miR-199a-3p, miR-214-3p). In this study, we verified the level of circulating miRNAs in the plasm of UM patients with different histologic types to find a circulating biomarker for the early diagnosis and prognostic evaluation of UM.

## Methods and Materials

### Selection of Candidate MicroRNAs

The selection of candidate microRNAs was based on the results of our previous experiments. The specimens of epithelioid cell melanoma (4 specimens) and spindle-shaped melanoma (4 specimens) of UM confirmed by histopathology and immunochemistry were collected in Beijing Tongren Hospital from March 2013 to October 2015. The expression profile of miRNA was assayed by miRNA array. Normal uveal specimens were obtained from 8 donors as controls. The differentially expressing miRNAs were screened by intergroup differential folds of ≥2. The microarray outcomes were validated by real-time quantitative PCR. The mutual upregulated miRNA in both spindle-shaped melanoma and epithelioid cell melanoma were miR-132-3p, miR-21-5p, miR-34a-5p, and miR-34b-5p, and mutual downregulated ones were miR-125b-2-3p, miR-126-3p, miR-199a-3p, and miR-214-3p (**Table 1**). Likely secreted miRNAs were selected from these miRNAs based on literature search.

**Table 1:** The results of real-time quantitative PCR performed on UM tissue samples.

miRNA	Spindle-shaped melanoma 2^-△△ct^(Mean ± SD)	*p* value	Epithelioid cell melanoma 2^-△△ct^(Mean ± SD)	*p* value
miR-132-3p	7.44 ± 1.98	<0.001	4.18 ± 0.99	<0.001
miR-21-5p	4.37 ± 1.02	0.049	3.41 ± 0.97	0.011
miR-34a-5p	8.93 ± 0.98	<0.001	9.2 ± 1.03	<0.001
miR-34b-5p	241.20 ± 7.02	<0.001	3.83 ± 0.54	<0.001
miR-125-2-3p	0.03 ± 0.01	<0.001	0.05 ± 0.01	<0.001
miR-126-3p	0.22 ± 0.01	0.002	0.15 ± 0.01	0.003
miR-199a-3p	0.25 ± 0.02	<0.001	0.12 ± 0.02	<0.001
miR-214-3p	0.12 ± 0.01	<0.001	0.02 ± 0.01	<0.001

### Serum Sampling

We collected 20 blood samples from UM patients who underwent eyeball enucleation in Beijing Tongren Hospital, Capital Medical University, Beijing, China, between December 2017 and October 2019. All blood samples were collected before treatment. As of the last follow-up, 20 patients with localized UM were in good survival conditions, and no melanoma metastasis was found. The blood of 10 healthy subjects was collected as a control. All healthy subjects have no history of tumors or long-term medication history before collecting blood samples.

To further evaluate the applicability of the biomarkers in patients with advanced UM, the blood samples of 5 patients with metastatic UM undergoing primary ocular treatment in Beijing Tongren Hospital were collected from January 1, 2020, to September 1, 2020, as a verification set. All metastatic tumors were identified by imaging and pathological examination. Blood samples were collected before all primary ocular treatments.

The fasting venous blood was taken as a blood sample. The blood sample was placed in a vacuum tube at room temperature for half an hour, and then it was centrifuged at 1,200 rpm for 15 min at 4°C to obtain the serum sample. Serum was divided into aliquots and stored at -80°C until analysis. All patients and healthy donors were informed of the purpose of the study and asked to sign an informed consent form. This study was reviewed by the Ethics Committee of Capital Medical University.

### Clinicopathological Data

**Figure 1:**
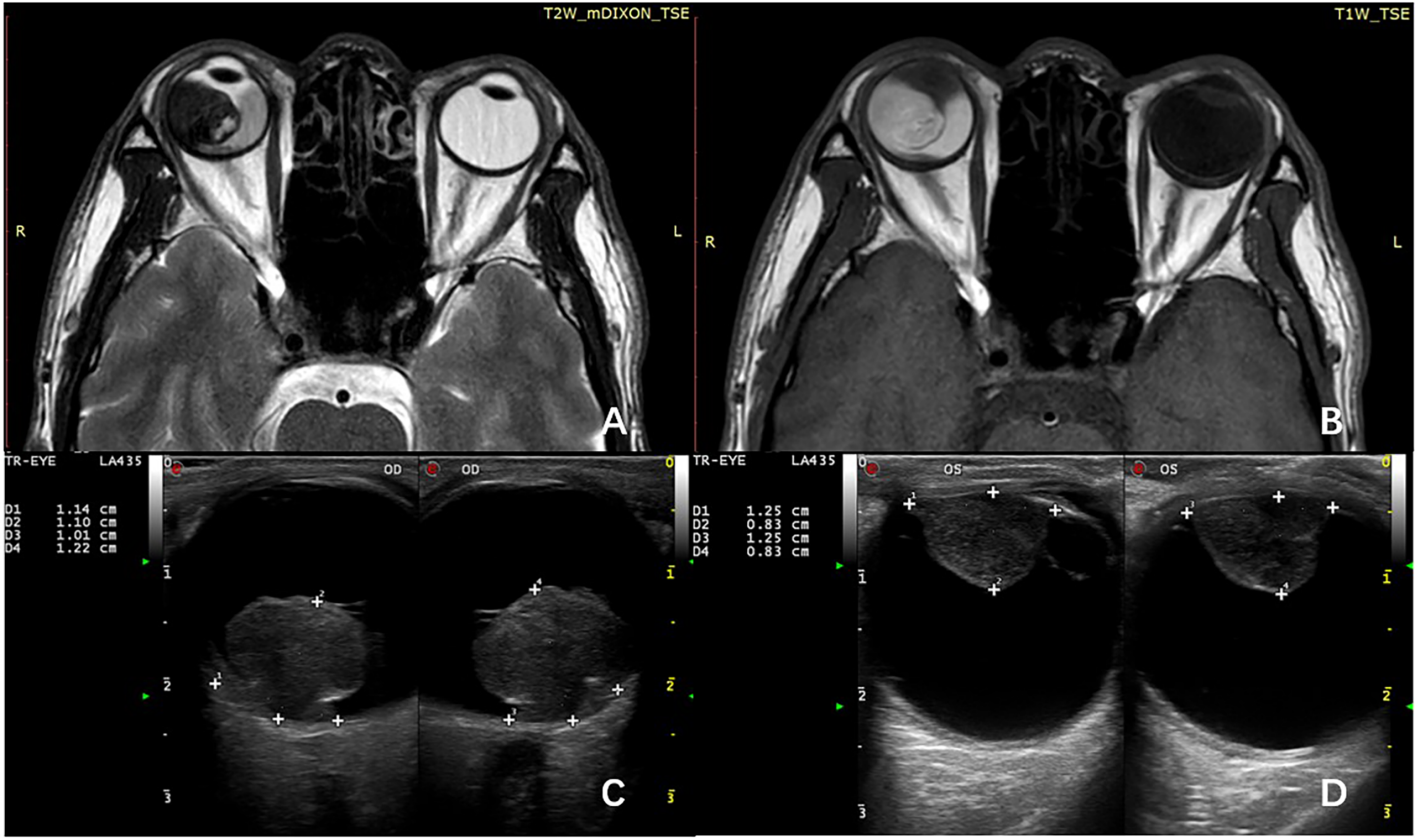
MRI shows the primary tumor has spread to the ciliary body on T1-weighted image **(A)** and T2-weighted image **(B)**. B-scan ultrasonography can roundly measure the size of choroid melanoma **(C)** and ciliary body melanoma **(D)**.

All patients underwent thorough ophthalmologic evaluations before enucleation to confirm the diagnosis of UM and confirm that they did not meet the indications for plaque radiotherapy or local resection. Histological examination was performed at the Unit of Pathology, Beijing Tongren Hospital, Capital Medical University, Beijing, China. The clinicopathological data include the age at diagnosis, sex, location of the tumor, the maximum diameter of the tumor, the height of the tumor (the size of tumor was measured after enucleation), tumor cell type, and extrascleral extension. All patients that underwent imaging examination including MRI and ultrasonography before enucleation, the tumor size, ciliary body involvement, and extraocular extension were initially assessed by imaging techniques (**Figure 1**), and pathological examination was employed for further assessment after enucleation.

### RNA Isolation

Total RNA enriched for small RNAs was isolated using miRcute Serum/Plasma Kit (Tiangen Biotech, Ltd., Beijing, China) from 200 μl of blood serum according to the manufacturer’s protocol. The concentration and purity of RNA were determined using the NanoDrop ND-2000 spectrophotometer (Thermo Scientific, Wilmington, DE, USA). The samples were either stored at –80°C or immediately processed.

### qRT-PCR Quantification of miRNA Expression in Serum

Hifair^®^ II 1st Strand cDNA Synthesis Kit (Yeasen Biotechnology Co., Ltd., Shanghai, China) was used for reverse transcription of 3 μl of RNA sample. Real-time PCR was performed using the Hieff^®^ qPCR SYBR Green Master Mix (Yeasen Biotechnology Co., Ltd., Shanghai, China). The 20-μl PCR reaction mixture included 10 μl Hieff^®^ qPCR SYBR Green Master Mix, 5 μl cDNA, and 1 μl of primer (0.5 μl forward primer and 0.5 μl reverse primer). Reactions were incubated in a 96-well optical plate at 95°C for 5 min, followed by 40 cycles at 95°C for 10 s, 60°C for 20 s, and 72°C for 20 s. All real-time PCR reactions were run in triplicates.

### Statistical Analysis

The average levels of all measured miRNAs were normalized by the use of U6 and subsequently analyzed by the 2−ΔCt method. If the CT value is greater than 35, the data are unreliable and will not be adopted. Statistical analyses were performed on GraphPad Prism version 6 (GraphPad software, USA). Sensitivity, specificity, and area under curve (AUC) for miRNA levels were determined using receiver operator characteristic (ROC) analysis. Student’s t test was adopted if the clinical-pathological data were normally distributed. We performed the Mann–Whitney-U-test and Fisher’s exact test when the data were not normally distributed.

## Results

### Clinicopathological Characteristics of Uveal Melanomas

A total of 20 patients with UM were included in the study, including 10 spindle-shaped melanomas and 10 epithelioid cell melanomas. In addition, the blood of 10 healthy donors was collected as a normal control (**Table 2**). The average age of healthy donors was 41 ± 5.2 years, the average age of patients with spindle-shaped melanoma was 44.7 ± 4.5 years at the time of diagnosis, and the average age of patients with epithelioid cell melanoma was 40.9 ± 7.8 years at the time of diagnosis. The proportion of men in the three groups was 50.0%. There were no significant differences in clinicopathological parameters between patients with epithelioid cell melanoma and patients with spindle-shaped melanoma, including average tumor diameter (t = 0.075, *p* = 0.941), average tumor height (t = 0.561, *p* = 0.582), and tumor location (*p* = 1.00), extrascleral extension (*p* = 1.00), or systemic metastasis (*p* = 1.00). The clinicopathological data of participants with metastatic UM are given in **Table 3**. Most of the patients are male (80%), and the histological type is mainly epithelioid cell melanoma (80%). The average age, average tumor diameter, and average tumor height of metastatic patients are 52.8 ± 12.6 years, 15.3 ± 3.7 mm, and 11.7 ± 2.0 mm, respectively.

**Table 2:** Data of participants in this study.

Variable	Healthy controls	Spindle-shaped melanoma	Epithelioid melanoma		*p* value
**Age (years)**	41 ± 5.2	44.7 ± 4.5	40.9 ± 7.8		
**Sex (male/female)**	5/5	5/5	5/5		
**Largest diameter (mm)**		10.9 ± 6.2	10.7 ± 6.8	t = 0.075	0.941
**Thickness (mm)**		8.2 ± 3.3	9.4 ± 5.7	t = 0.561	0.582
**Location (n, %)**					
Choroid		9 (90.0)	8 (80.0)		1.000
Ciliary body		1 (10.0)	2 (20.0)		
**Extrascleral extension (n, %)**					
Yes		0 (0.0)	0 (0.0)		1.000
No		10 (100.0)	10 (100.0)		
**Metastasis (n, %)**					
Yes		0 (0.0)	0 (0.0)		
No		10 (100.0)	10 (100.0)		

**Table 3:** Data of patients with metastatic UM.

Sex	Age	Location	Thickness (mm)	Largest diameter (mm)	Cell type	Extrascleral extension	Metastasis site
Female	49	Choriod	10.7	11.7	Spindle	No	Bone
Male	61	Choriod	9.8	19.1	Epithelioid	No	Liver
Male	56	Choriod	14	16.2	Epithelioid	No	Liver
Male	33	Choriod	10.4	11.2	Epithelioid	No	Liver
Male	65	Choriod	13.8	18.3	Epithelioid	No	Liver

### Imaging Features of Uveal Melanomas

**Figure 2:**
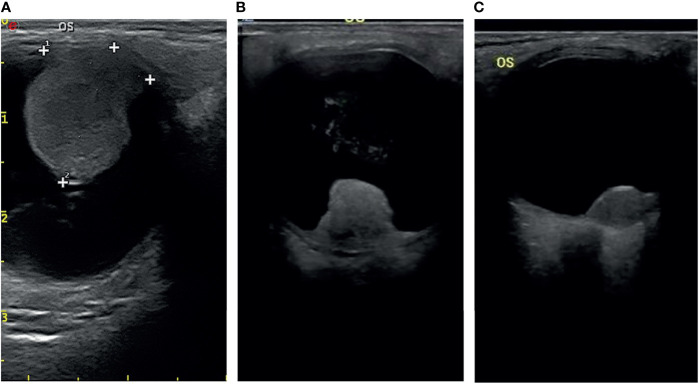
The typical pattern of UM in B-scan ultrasonography **(A)**, mushroom configuration **(B)**, and acoustic hollowness **(C)** choroidal excavation.

**Figure 3:**
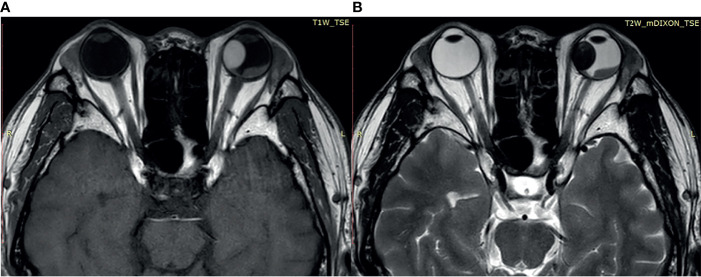
**(A)** The tumor demonstrates a relatively bright signal on the T1-weighted image. **(B)** The tumor demonstrates a relatively low signal on the T2-weighted image.

Most of the UMs in this study show a typical pattern in B-scan ultrasonography, which appears as mushroom configuration, acoustic hollowness, and choroidal excavation (**Figure 2**). They also show a fairly typical pattern on T1-weighted images, demonstrating a bright signal relative to the vitreous compared with T2-weighted images, which provide a low tumor signal compared with the vitreous (**Figure 3**).

### miRNA Expression in Serum of UM Patients

**Figure 4:**
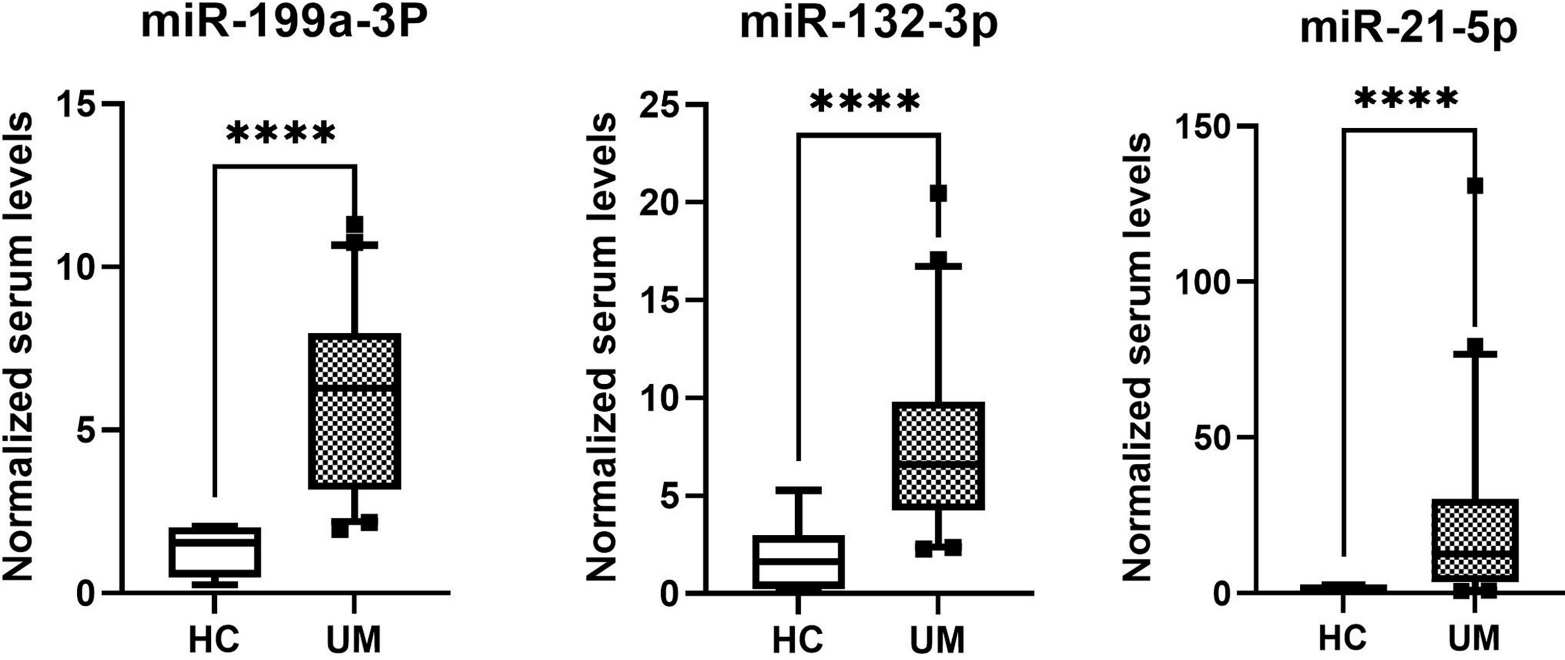
MicroRNAs with significant different levels in serum of uveal melanoma (UM) patients and healthy controls (HC), ****p < 0.0001.

**Figure 5:**
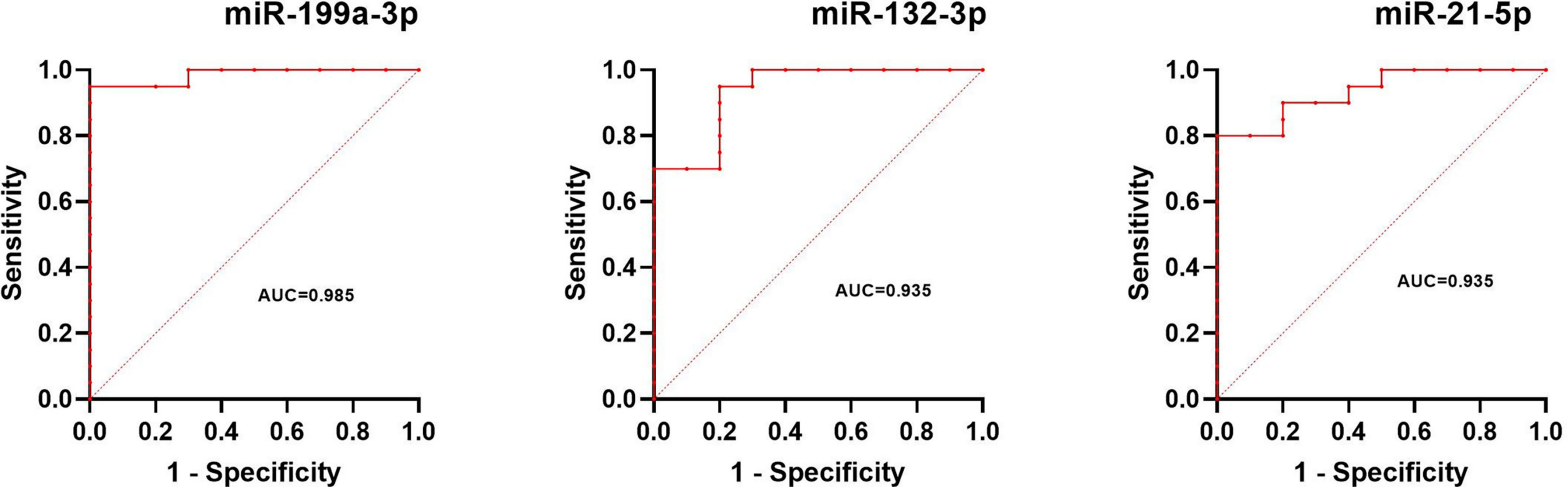
Results of ROC analysis of miR-199a-3p, miR-132-3p, and miR-21-5p between UM patients and healthy controls.

The normalized serum levels of miRNAs in UM patients and healthy controls are given in **Table 4**. The CT value of miR-34a-5p is greater than 35. The miR-21-5p (Z = 3.83, *p* < 0.0001), miR-199a-3p (Z = 4.27, *p* < 0.0001), and miR-132-3p (Z = 3.83, *p* < 0.0001) tested had significantly higher levels in serum of UM patients compared to healthy donors (**Figure 4**). The ROC curve was used to evaluate the diagnostic performance of the above three miRNAs (**Figure 5**), and the results showed that miR-199a-3p had the highest diagnostic performance, with an AUC of 0.985; when the normalized serum level of miR-199a-3p is 2.115, the sensitivity is 95% and the specificity is 100%.

**Table 4:** The normalized serum levels of miRNAs in UM patients and healthy controls.

miRNAs	HC M (P25, P75)	UM M (P25, P75)	Wilcoxon Mann–Whitney	
			Z value	*p* value
miR-21-5p	0.78 (0.57, 2.00)	11.72 (3.64, 30.62)	3.83	<0.0001
miR-199a-3p	1.54 (0.48, 2.02)	6.28 (3.17, 7.98)	4.27	<0.0001
miR-132-3p	1.63 (0.25, 2.99)	6.59 (4.25, 9.81)	3.83	<0.0001
miR-126-3p	1.06 (0.47, 2.19)	1.44 (1.17, 2.19)	0.88	0.379
miR-214-3p	0.78 (0.68, 1.46)	0.55 (0.38, 0.76)	2.42	0.015

**Figure 6:**
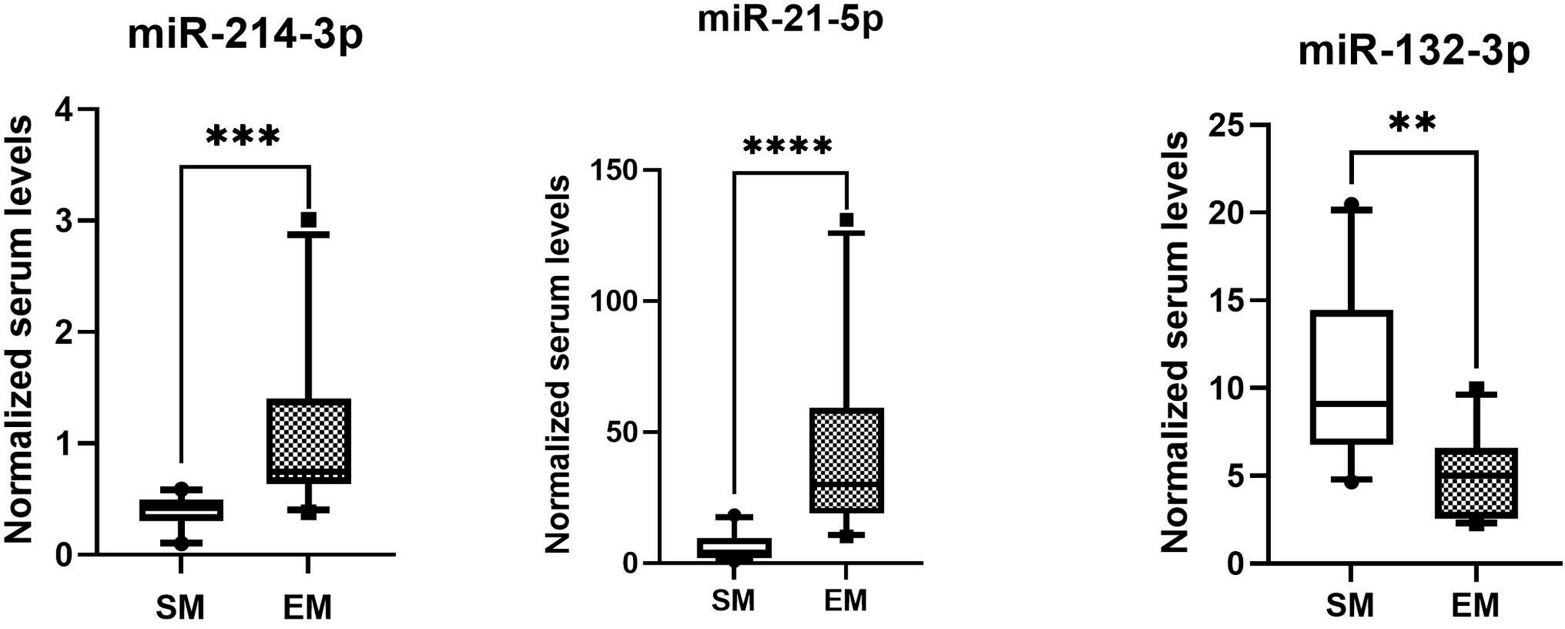
MicroRNAs with significant difference in serum of spindle melanoma (SM) and epithelioid cell melanoma (EM) ****p < 0.0001, ***p < 0.001; **p < 0.01.

**Figure 7:**
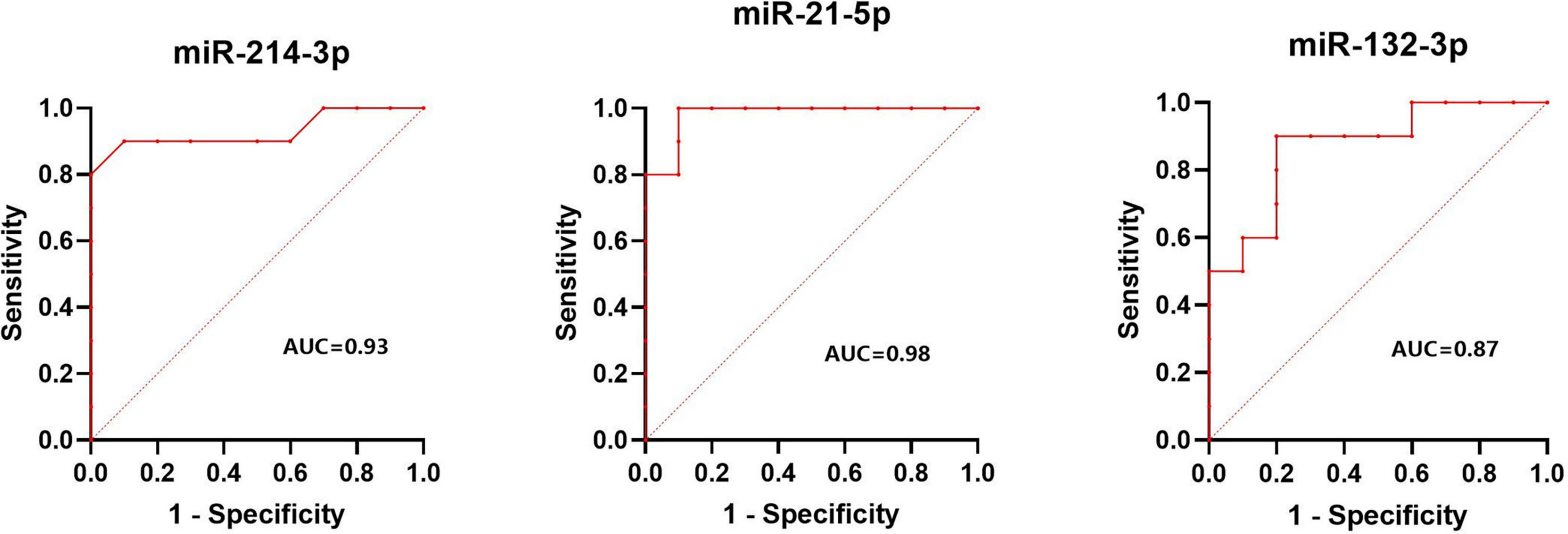
Results of ROC analysis of miR-214-3p, miR-21-5p, and miR-132-3p between spindle melanoma and epithelioid cell melanoma.

The normalized serum levels of miRNAs in spindle-shaped melanoma and epithelioid cell melanoma are given in **Table 5**. The CT value of miR-34a-5p is greater than 35. The level of miR-132-3p (Z = 2.80, *p* < 0.01), miR-214-3p (Z = 3.25, *p* < 0.001), and miR-21-5p (Z = 3.63, *p* < 0.0001) in serum had a significant difference between spindle-shaped melanoma and epithelioid cell melanoma (**Figure 6**). The ROC curve was used to evaluate the ability to distinguish histologic types of the above three miRNAs (**Figure 7**), and the results showed that miR-21-5p had the best performance, with an AUC of 0.98. When the normalized serum level of miR-21-5p is 9.97, the sensitivity is 100% and the specificity is 90%.

**Table 5:** The normalized serum levels of miRNAs in spindle-shaped melanoma and epithelioid cell melanoma.

miRNAs	Spindle-shaped melanoma M (P25, P75)	Epithelioid cell melanoma M (P25, P75)	Wilcoxon Mann–Whitney	
			Z value	*p* value
miR-21-5p	3.97 (1.76, 9.45)	29.98 (18.84, 59.17)	3.63	<0.0001
miR-199a-3p	6.80 (4.81, 9.88)	3.65 (2.34, 7.30)	4.27	0.07
miR-132-3p	9.1 (6.77, 14.45)	5.02 (2.55, 6.57)	2.80	<0.01
miR-126-3p	1.30 (0.96, 1.82)	1.57 (1.14, 2.65)	0.8	0.236
miR-214-3p	0.42 (0.30, 0.50)	0.75 (0.64, 1.40)	3.25	<0.001

**Figure 8:**
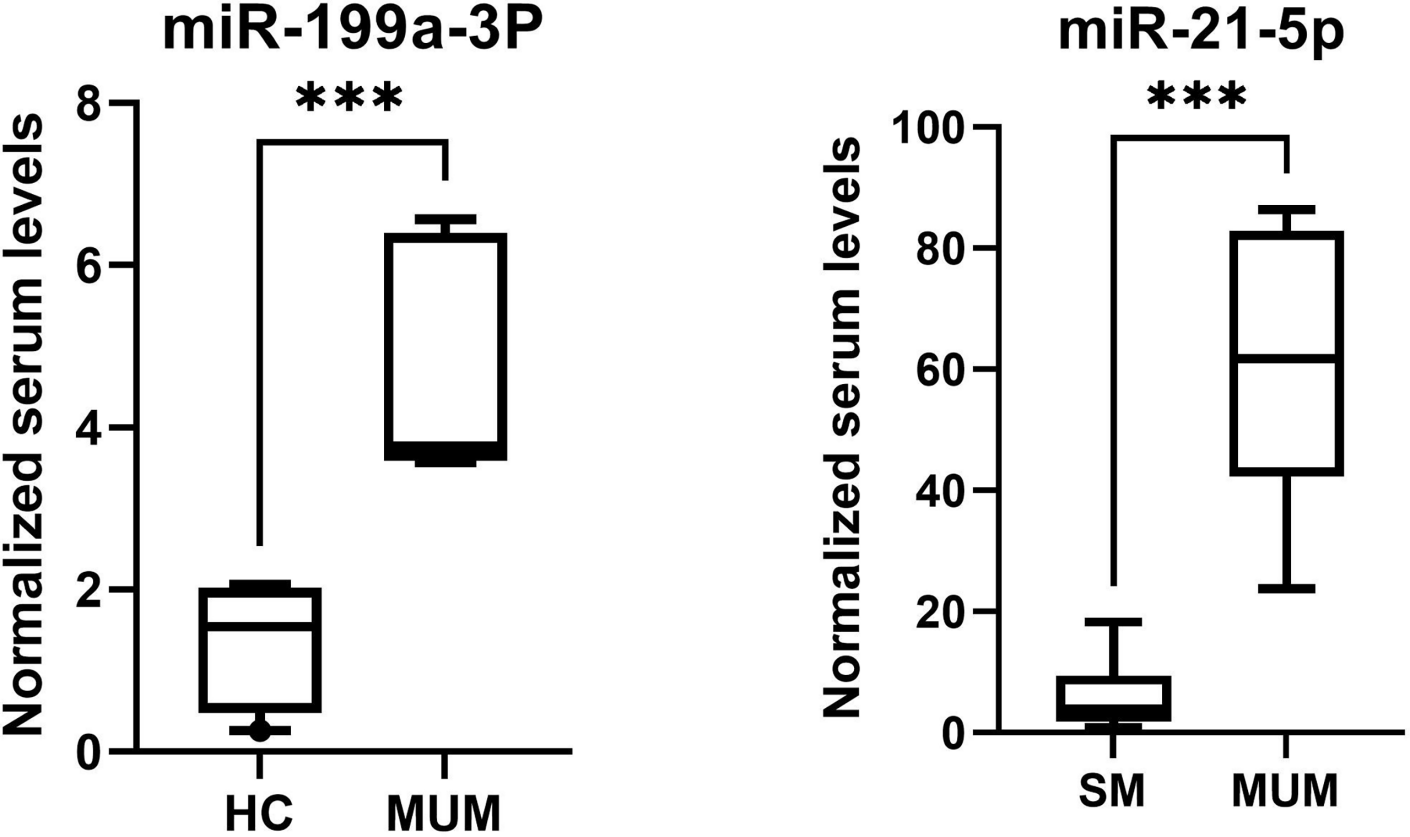
The higher levels of miR-199a-3p of metastatic UM patients in comparison to healthy controls (HC) and higher levels of miR-21-5p of metastatic UM patients (MUM) in comparison to patients with localized spindle shaped melanoma (SM). ***p < 0.001.

As is depicted in **Figure 8**, significantly higher levels of miR-21-5p (Z = 2.51, *p* < 0.001) were found in serum of metastatic UM patients compared to patients with localized spindle-shaped melanoma. Moreover, beyond that, significantly higher levels of miR-199a-3p (Z = 3.07, *p* < 0.001) were detected in serum of metastatic UM patients compared to healthy donors.

## Discussion

In this study, we found that the serum levels of miR-199a-3p, miR-21-5p, and miR-132-3p are significantly increased in UM patients compared with healthy controls, and miR-199a-3p has the best diagnostic performance. We also found that the serum levels of miR-21-5p, miR-132-3p, and miR-214-3p differ significantly between epithelioid cell melanoma patients and spindle-shaped melanoma patients, and miR-21-5p has the best performance to distinguish histologic types. The diagnosis of UM is mainly primary diagnosis with a relatively high accuracy in the specialized ophthalmic center. In order to confirm the diagnosis of UM with non-invasive methods, a variety of imaging techniques can be employed (20, 21). Ultrasonography and MRI play an important role in the diagnosis of UM, especially in eyes with opaque media (22). Fundus fluorescein angiography (FFA) evaluates the intrinsic tumor vasculature which is useful in the differential diagnosis of choroidal lesions demonstrating different vascular patterns. Optical coherence tomography (OCT) is particularly useful to image small melanomas which are 3 mm thick or less, and it is valuable for the early detection of UM, especially when considering factors for distinguishing a choroidal naevus from a UM (23). However, the diagnosis with imaging techniques is mainly based on the morphological features of UM, which is not obvious in the early stage (24, 25). Our study found that circulating miR-199a-3p can be used to effectively diagnose UM. Compared with healthy controls, miR-199a-3p, miR-21-5p, and miR-132-3p are significantly increased in the serum of UM patients, and miR-199a-3p has the best diagnostic performance. 20 patients with localized UM in this study were in good survival conditions after enucleation; the serum level of miR-199a-3p can increase in the early stage of UM. With the significantly higher levels of miR-199a-3p in serum of metastatic UM patients, the serum level of miR-199a-3p can also increase in the advanced UM. Compared with tumor biopsy, blood collection carries a low risk of procedure-related complications. MiR-199a-3p is a new, non-invasive, and quantitative biomarker that can be used for the early diagnosis of UM. Many studies have demonstrated that miR-199a-3p acts as antioncogene in a variety of tumors (26–29). Worely et al. (30) analyzed the miRNA expression patterns in 24 specimens of UM and miR-199a was significantly upregulated. However, in our previous study, the level of miR-199a-3p was significantly downregulated on formalin-fixed, paraffin-embedded UM samples compared with choroid tissue. On the one hand, the sample size was relatively small in our previous study, which may lead to sampling errors. On the other hand, the circulating miRNAs do not completely reflect the tumor cellular profile (31). The exact molecular mechanisms about how tumor cells sort miRNAs to the blood remain quite elusive (32), and the role of miR-199a-3p in the UM and its underlying mechanism still need further study.

The tumor cells have disseminated in the very early stage, as primary ocular treatment of the eye with a UM does not prevent the development of these metastases (1). As a result, it is of great significance to assess the prognosis followed by the development of the best treatment. The prognosis of UM is related to histopathologic features, tumor size and location, extraocular extension, and genetic changes (33). Chromosomal alterations like monosomy 3 and gain of chromosome 8q and mutations like *BAP1*, *SF3B1*, and *EIF1AX* are strongly associated with high metastatic risk and poor survival (34). The abovementioned genetic changes have a clinical value as prognostic marker; however, the genetic testing is time-consuming and labor-intensive and the sample needs to be obtained by invasive biopsy, which makes it difficult to be applied in clinical practice. Tumor size, ciliary body involvement and extraocular extension are also important negative prognostic factors. These negative prognostic factors enable imaging examinations to evaluate the prognosis of UM at a limited level. Although it may cause radiation damage to patients, positron emission tomography/computed tomography (PET/CT) may have a role in predicting the prognosis of UM, since the standardized uptake value (SUV) may be correlated with risk factors for metastasis, like the proportion of epithelioid cell and tumor size (35). Unfortunately, imaging examinations cannot diagnose UM in an early stage and quantitatively evaluation of the prognosis of UM is either not possible. Circulating microRNA is an ideal blood-based biomarker. Studies have shown that the microRNA expression profiling of UM patients is significantly different from that of healthy controls and also has significant differences in UM with different prognosis (19, 30). Histologic types are the most commonly used method to assess the prognosis of patients after enucleation in routine clinic practice. Three histologic types of uveal melanoma include spindle-shaped melanoma, mixed melanoma, and epithelioid cell melanoma. Epithelioid melanoma is more malignant than the other two types, and the higher the proportion of epithelial cells, the more malignant the UM (36, 37). We found that the level of miR-21-5p in the blood of epithelioid cell melanoma patients was significantly increased compared with spindle-shaped melanoma. In our previous study, miR-21-5p was significantly upregulated in epithelioid cell melanoma tissue and spindle-shaped melanoma tissue, which was consistent with the significant upregulation of miR-21-5p in the serum of UM patients. MiR-21-5p acts as an oncogene in many tumors, and it has been shown to be involved in the epithelial–mesenchymal transition necessary for metastasis (38–40). Other research showed that the level of miR-21-5p is significantly increased in high-risk UM tissues, which implies that it can be responsible for UM metastasis (41). Since one miRNA can target a large number of genes and most studies use target genes identified by online prediction algorithms, it is important to perform validation experiments in UM cell lines (12, 31, 41). The validation experiment of miR-21 has already been performed in UM cell lines. It may inhibit the expression of wild-type p53 genes and increase the expression of *LASP1* at the mRNA level and protein level, which promotes the proliferation and metastasis of UM (42). Our findings may provide a quantitative prognostic biomarker for UM patients.

Several limitations of this study should be noted. All our patients were from the same ophthalmic center, which did not cover persons from multiple ethnic and regional populations. Another limitation of this study is the small sample size of participants with UM. Prospective studies with a large sample size and comparing varying ethnic groups are required to further evaluate the performance of the biomarkers.

## Data Availability Statement

The original contributions presented in the study are included in the article Supplementary Material. Further inquiries can be directed to the corresponding author.

## Ethics Statement

The studies involving human participants were reviewed and approved by the Ethics Committee of Capital Medical University. The patients/participants provided their written informed consent to participate in this study.

## Author Contributions

W-DZ, LS, and W-BW contributed to study conception. W-DZ, H-TW, R-HZ, H-YL, X-HS, and Y-FL contributed to collect the data of healthy controls and UM patients and get blood samples. W-DZ, LS, and LD performed the laboratory operation. W-DZ and LS wrote the initial draft of the manuscript. LS and LD contributed to editing of the present version. W-BW commented on the manuscript and gave final approval of the version to be published. All authors contributed to the article and approved the submitted version.

## Funding

This study was supported by the Capital Health Research and Development of Special (2020-1-2052); Science & Technology Project of Beijing Municipal Science & Technology Commission (Z201100005520045, Z181100001818003); the Priming Scientific Research Foundation for the Junior Researcher in Beijing Tongren Hospital, Capital Medical University (No.2018-YJJ-ZZL-045); and Beijing Outstanding Talent Project Youth Backbone Foundation (2018000021469G207).

## Conflict of Interest

The authors declare that the research was conducted in the absence of any commercial or financial relationships that could be construed as a potential conflict of interest.

## Publisher’s Note

All claims expressed in this article are solely those of the authors and do not necessarily represent those of their affiliated organizations, or those of the publisher, the editors and the reviewers. Any product that may be evaluated in this article, or claim that may be made by its manufacturer, is not guaranteed or endorsed by the publisher.
